# Complete Genome Sequence of Pasteurella multocida Sequence Type 394, Isolated from a Case of Bovine Respiratory Disease in Australia

**DOI:** 10.1128/mra.00890-21

**Published:** 2022-03-02

**Authors:** P. Roy Chowdhury, Tamara Alhamami, Henrietta Venter, Tania Veltman, Mandi Carr, Joanne Mollinger, Darren J. Trott, Steven P. Djordjevic

**Affiliations:** a The ithree Institute, University of Technology Sydney, Ultimo, Sydney, Australia; b Australian Centre for Antimicrobial Resistance Ecology, School of Animal and Veterinary Sciences, University of Adelaide, Roseworthy, Australia; c Clinical Health Sciences, University of South Australia, Adelaide, Australia; d Biosecurity Sciences Laboratory, Department of Agriculture and Fisheries, Health and Food Sciences Precinct, Coopers Plains, Queensland, Australia; Loyola University Chicago

## Abstract

Here, we present the completely closed genome sequence of Pasteurella multocida 17BRD-035, a bovine respiratory disease (BRD) pathogen from Queensland, Australia, with genes that confer resistance to β-lactams, tilmicosin, and tetracycline. It consists of a single 2,624,884-bp chromosome and an average GC content of 40.23% and belongs to the newly described Rural Industries Research and Development Corporation (RIRDC) sequence type 394.

## ANNOUNCEMENT

We present a completely closed genome sequence of a recently catalogued multilocus sequence type (MLST) variant, sequence type 394 (ST394), of Pasteurella multocida (strain 17BRD-035) for the RefSeq database. The ST394 designation is based on housekeeping genes used for the P. multocida Rural Industries Research and Development Corporation (RIRDC) MLST scheme (1) available via pubMLST. Using the multihost sequence typing scheme developed for P. multocida (2), 17BRD-035 represents ST159. P. multocida is one of the most frequently isolated bacterial pathogens associated with bovine respiratory disease (BRD) and is increasingly associated with antimicrobial resistance in the feedlot cattle industry, both globally and within Australia (3).

Strain 17BRD-035 was isolated from a diagnostic submission (lung swab from a BRD-affected animal collected postmortem) in Queensland, Australia, in 2017 (3). The strain was maintained on sheep blood agar for routine microbiological assays and grown in brain heart infusion broth for DNA extraction. Details of DNA preparation steps, sequencing kits and software used for bioinformatic analyses, presented in Table 1, are as follows: DNA extraction for Illumina HiSeq (Bioline isolate II genomic DNA extraction kit), HiSeq library preparation (Illumina Nextera XT), Nanopore DNA preparation (XS buffer lysis followed by phenol-chloroform purification [4]), Nanopore sequencing (rapid barcoding sequencing kit [SQK-RBK004], R9.4.1 flow cell [FLO-MIN106]), Nanopore base calling (Guppy [5]), Quality control (QC; FastQC v0.11.9 [6], fastp v0.22.0 [7], and pycoQC 2.5.0.23 [8]), and QC data collection (MultiQC v1.9 [9]). Unless otherwise specified, default parameters were used for all software tools. The genome was assembled *de novo* with the Unicycler v0.4.8 hybrid assembly (10) protocol utilizing both Illumina short read and Nanopore sequence data generated in-house at the University of Technology Sydney sequencing facility. The genome was assembled into a single circular chromosome 2,624,884 bp in length and with an average GC content of 40.23% (GenBank accession CP082272) (Table 1). Preliminary annotations using Prokka v1.7 (11) revealed the presence of 2,547 coding DNA sequences (CDS) and 23 rRNA and 66 tRNA gene sequences in the genome. It contained β-lactamase-encoding *bla*_ROB-1_ and tetracycline resistance-conferring *tetR*/*tetH* genes, as well as a number of genes conferring resistance to heavy metals (*corC*, magnesium and cobalt efflux; *mco*, copper oxidase family of genes which confer copper resistance). In addition, the genome contains genes which encode MacA-MacB proteins which constitute a multidrug efflux pump and has been associated with variable macrolide resistance in other Gram-negative bacteria (12–14). Overexpression of these antibiotic efflux pumps may contribute to the nonsusceptibility to tilmicosin recorded by the strain.

**TABLE 1:** Reads generated by sequencing platforms and assembly statistics of the closed genome

Parameter	Data
Raw sequence reads	
Illumina paired-end read length (nt)	150
No. of Illumina left reads (R1)	1,872,270
No. of Illumina right reads (R2)	1,872,270
Properly paired Illumina reads (%)	94.91
No. of Nanopore reads (total)	145,043
No. of Nanopore reads (filtered)	143,212
Assembly statistics^*a*^ for the closed genome (CP082272)	
No. of contigs	1
Total length of the genome (bp)	2,624,884
Total no. of reads used	3,301,309
GC content (%)	40.23
Mapped reads (%)	99.65
Avg. coverage depth (×)	344
*N*_50_ for the hybrid assembly (bp)	2,624,884

a Assembled using Unicycler hybrid assembler.

**FIG 1:**
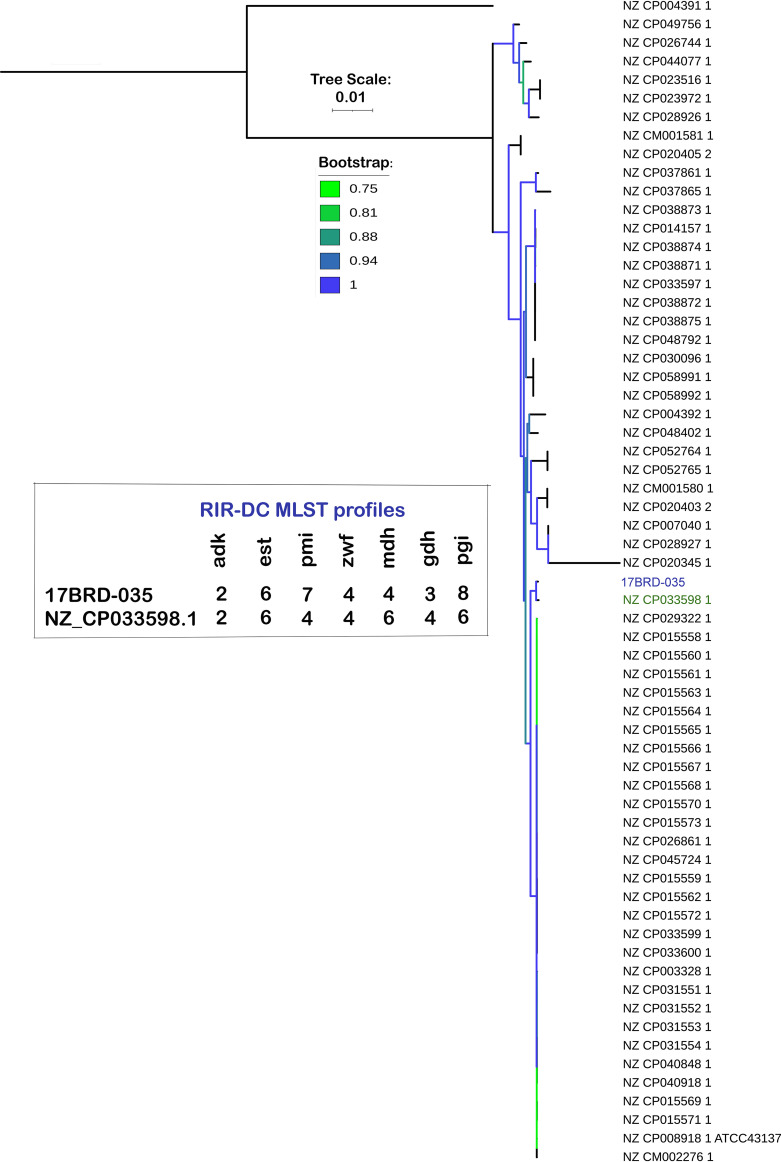
A midpoint-rooted phylogenetic tree of 17BRD-035 and 81 other genomes downloaded from the GenBank RefSeq database constructed using PhyloSift (https://github.com/gjospin/PhyloSift). The inset shows the RIRDC-MLST profile of 17BRD-035 (ST395) and the most closely related genome in the database NZ_CP033598.1 (ST7).

Due to the lack of completely closed ST394 genomes in the GenBank RefSeq database, we conducted a phylogenetic analysis of the 17BRD-035 genome with 81 finished genomes in the RefSeq database (accessed on 2 August 2021) using PhyloSift v20141105 (15). 17BRD-035 is most closely related to P. multocida strain CQ7 (Fig. 1), GenBank accession number NZ_CP033598.1. This strain was obtained from bovine pneumonic lung tissue originating from Chongqing, China, in 2013. A pairwise BLAST alignment of 17BRD-035 with CQ7 indicated 99.62% nucleotide identity over 83% of the query sequence. Although both 17BRD-035 and CQ7 belong to capsular serotype A (identified in-house on ABRicate v1.0.1 [https://github.com/tseemann/abricate] using a manually curated database following a strategy published by Peng et al. 2019 [16]), the CQ7 genome aligns with ST7 of the RIRDC-MLST scheme. ST7 differs from ST394 in the sequences of *pmi*, *mdh*, *gdh*, and *pgi* genes.

### Data availability.

The complete genome sequence of P. multocida 17BRD-035 has been deposited in GenBank under accession number CP082272 and BioSample number SAMN21014890 under BioProject PRJNA758188. Reads for the Hi-Seq run (SRR17163532) can be accessed in GenBank using accession number SRX13347427. Reads from the Oxford Nanopore run (SRR17853486) can be accessed in GenBank using accession number SRX14014314.
